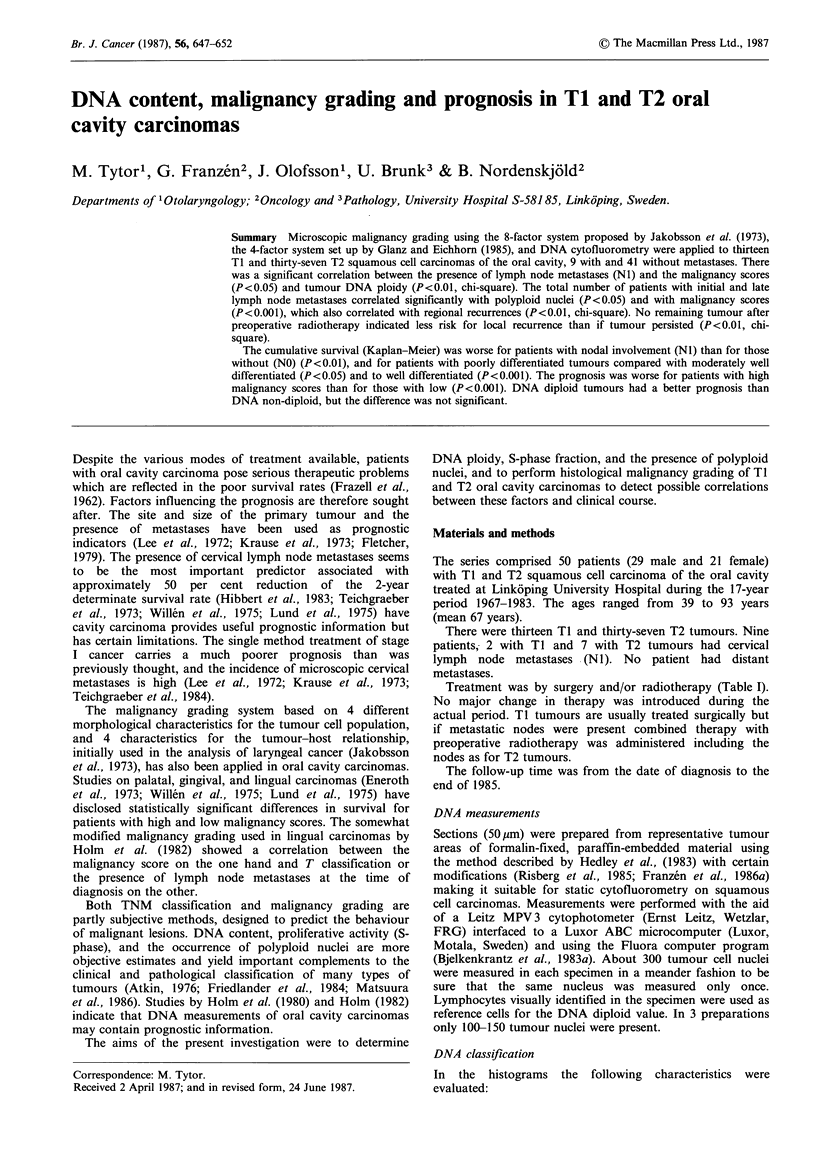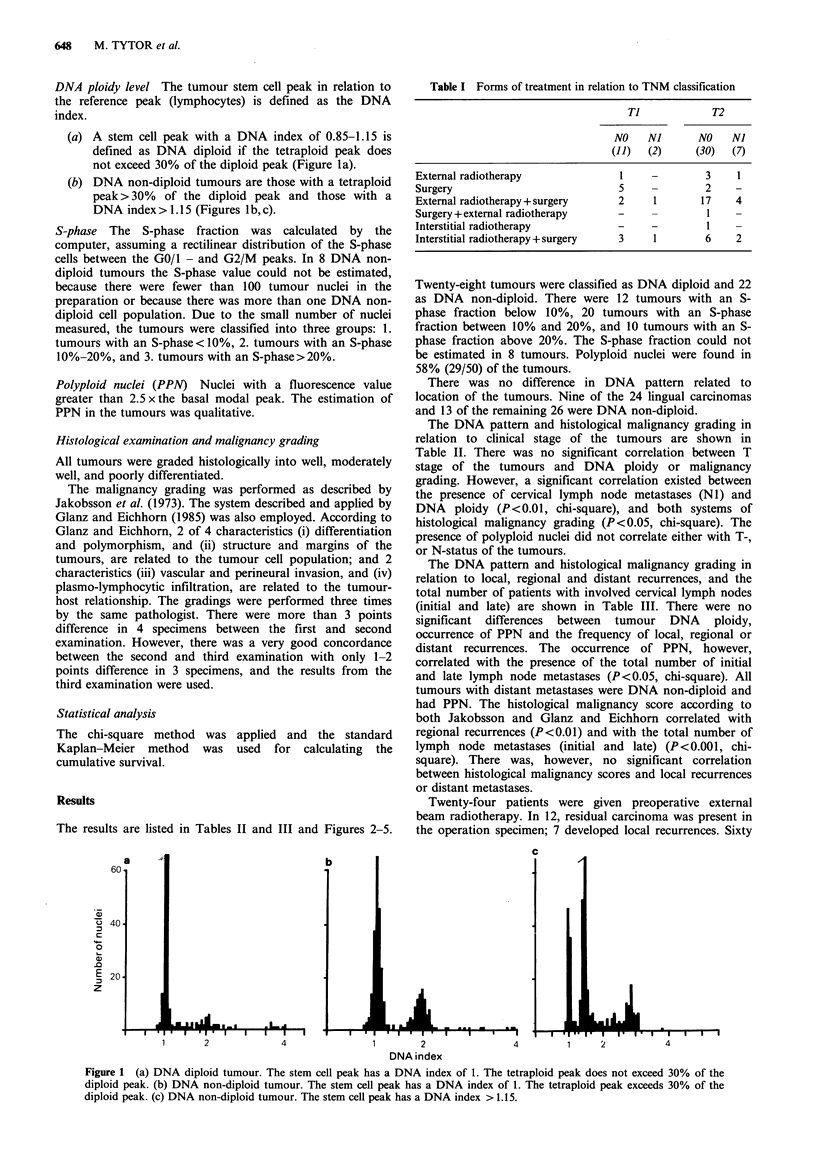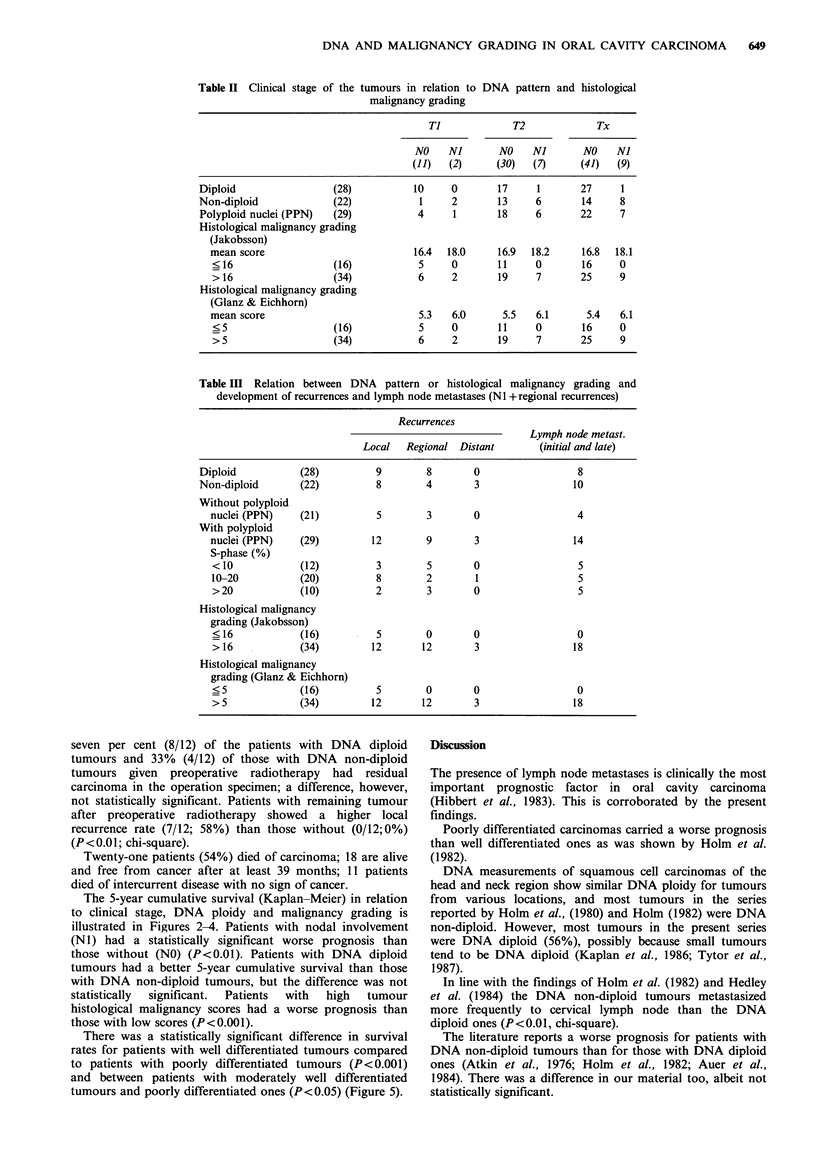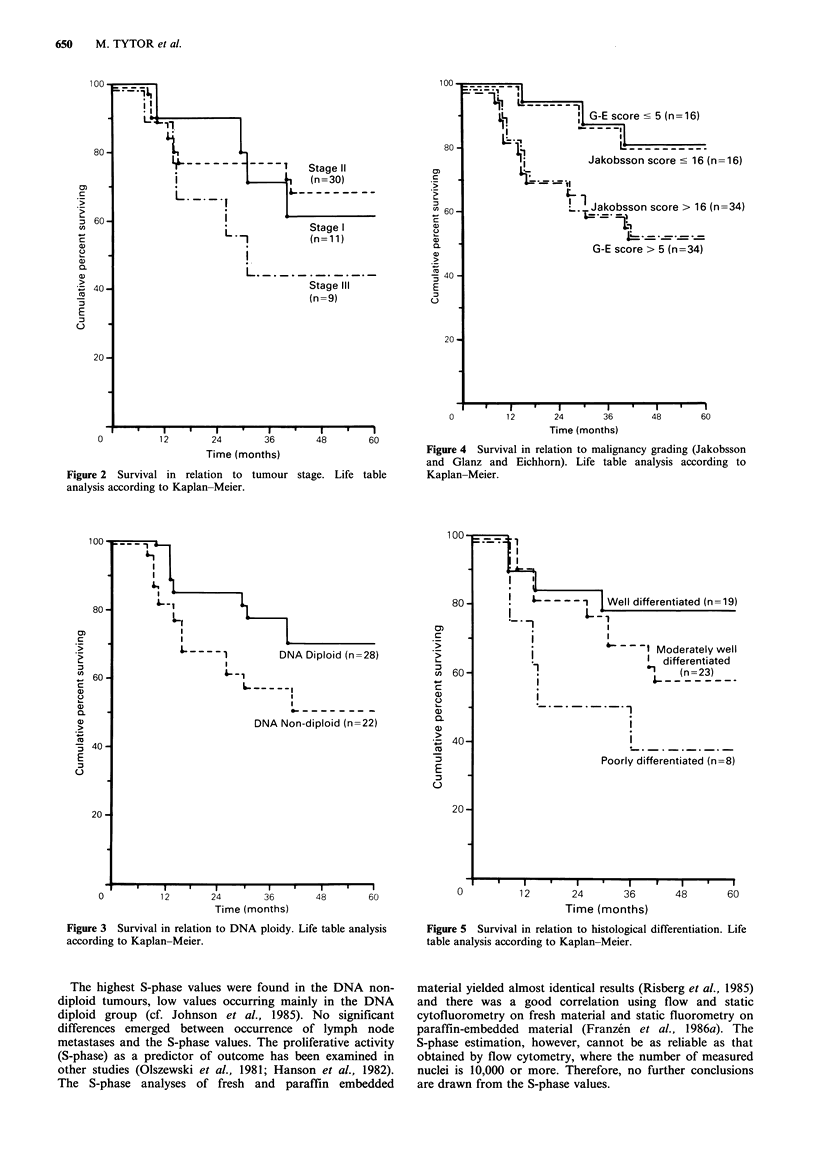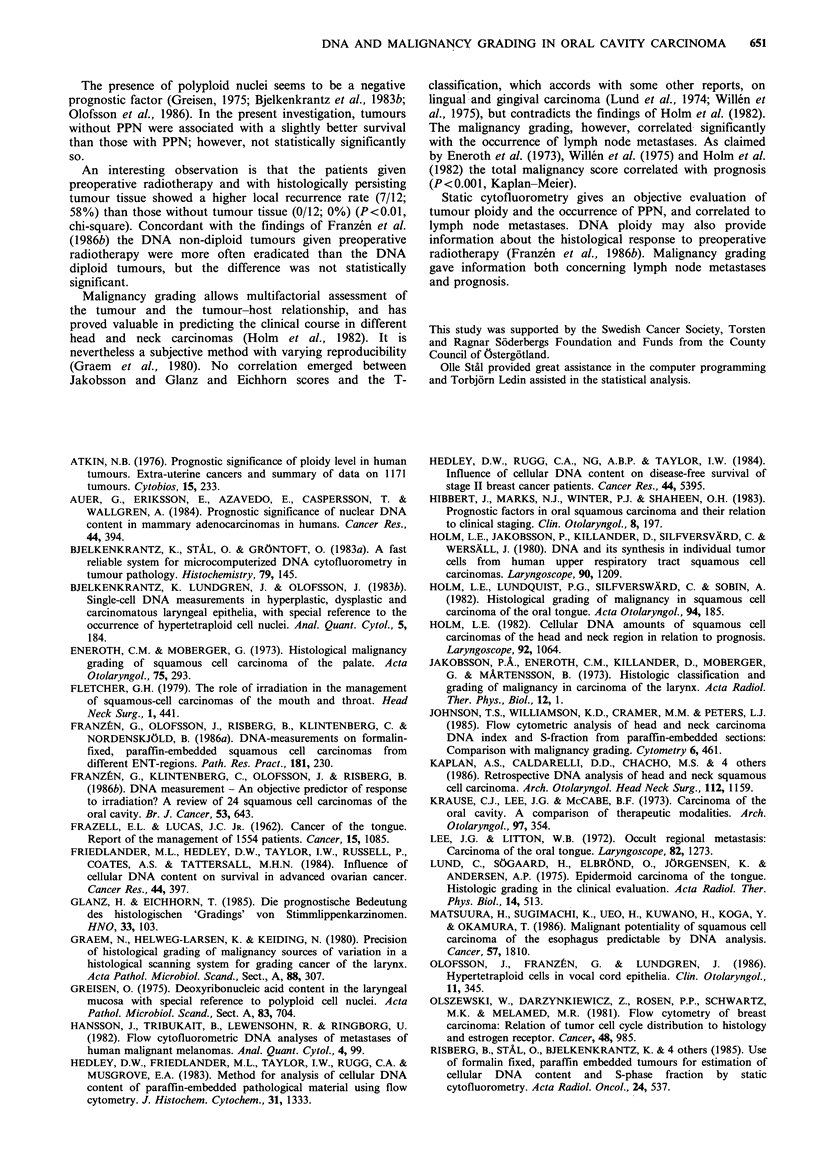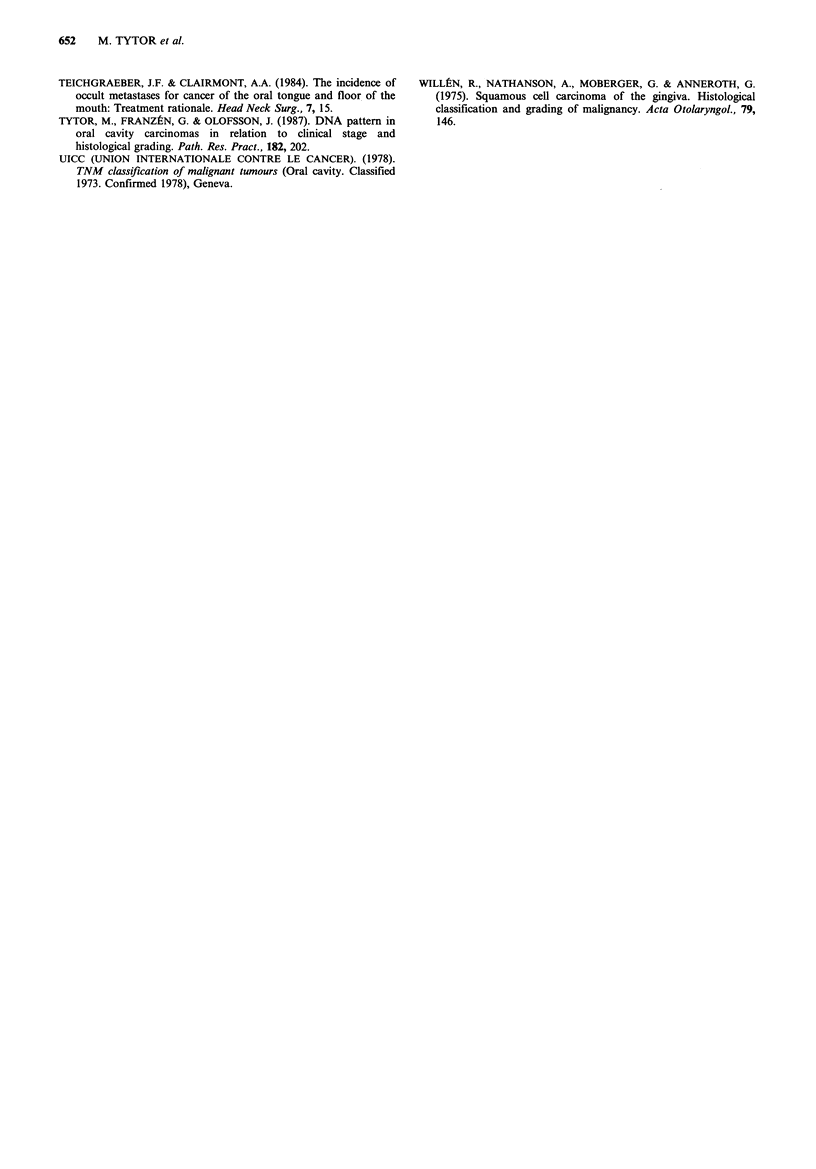# DNA content, malignancy grading and prognosis in T1 and T2 oral cavity carcinomas.

**DOI:** 10.1038/bjc.1987.260

**Published:** 1987-11

**Authors:** M. Tytor, G. Franzén, J. Olofsson, U. Brunk, B. Nordenskjöld

**Affiliations:** Department of Otolaryngology, University Hospital, Linköping, Sweden.

## Abstract

Microscopic malignancy grading using the 8-factor system proposed by Jakobsson et al. (1973), the 4-factor system set up by Glanz and Eichhorn (1985), and DNA cytofluorometry were applied to thirteen T1 and thirty-seven T2 squamous cell carcinomas of the oral cavity, 9 with and 41 without metastases. There was a significant correlation between the presence of lymph node metastases (N1) and the malignancy scores (P less than 0.05) and tumour DNA ploidy (P less than 0.01, chi-square). The total number of patients with initial and late lymph node metastases correlated significantly with polyploid nuclei (P less than 0.05) and with malignancy scores (P less than 0.001), which also correlated with regional recurrences (P less than 0.01, chi-square). No remaining tumour after preoperative radiotherapy indicated less risk for local recurrence than if tumour persisted (P less than 0.01, chi-square). The cumulative survival (Kaplan-Meier) was worse for patients with nodal involvement (N1) than for those without (N0) (P less than 0.01), and for patients with poorly differentiated tumours compared with moderately well differentiated (P less than 0.05) and to well differentiated (P less than 0.001). The prognosis was worse for patients with high malignancy scores than those with low (P less than 0.001). DNA diploid tumours had a better prognosis than DNA non-diploid, but the difference was not significant.


*Br. J. Cancer* (1987), **56**, 647–652

© The Macmillan Press Ltd., 1987

### DNA content, malignancy grading and prognosis in T1 and T2 oral cavity carcinomas

M. Tytor[1], G. Franzén[2], J. Olofsson[1], U. Brunk[3] & B. Nordenskjöld[2]

*Departments of [1]Otolaryngology; [2]Oncology and [3]Pathology, University Hospital S-581 85, Linköping, Sweden.*

**Summary** Microscopic malignancy grading using the 8-factor system proposed by Jakobsson *et al.* (1973), the 4-factor system set up by Glanz and Eichhorn (1985), and DNA cytofluorometry were applied to thirteen T1 and thirty-seven T2 squamous cell carcinomas of the oral cavity, 9 with and 41 without metastases. There was a significant correlation between the presence of lymph node metastases (N1) and the malignancy scores ($P<0.05$) and tumour DNA ploidy ($P<0.01$, chi-square). The total number of patients with initial and late lymph node metastases correlated significantly with polyploid nuclei ($P<0.05$) and with malignancy scores ($P<0.001$), which also correlated with regional recurrences ($P<0.01$, chi-square). No remaining tumour after preoperative radiotherapy indicated less risk for local recurrence than if tumour persisted ($P<0.01$, chi-square).

The cumulative survival (Kaplan–Meier) was worse for patients with nodal involvement (N1) than for those without (N0) ($P<0.01$), and for patients with poorly differentiated tumours compared with moderately well differentiated ($P<0.05$) and to well differentiated ($P<0.001$). The prognosis was worse for patients with high malignancy scores than for those with low ($P<0.001$). DNA diploid tumours had a better prognosis than DNA non-diploid, but the difference was not significant.

Despite the various modes of treatment available, patients with oral cavity carcinoma pose serious therapeutic problems which are reflected in the poor survival rates (Frazell *et al.*, 1962). Factors influencing the prognosis are therefore sought after. The site and size of the primary tumour and the presence of metastases have been used as prognostic indicators (Lee *et al.*, 1972; Krause *et al.*, 1973; Fletcher, 1979). The presence of cervical lymph node metastases seems to be the most important predictor associated with approximately 50 per cent reduction of the 2-year determinate survival rate (Hibbert *et al.*, 1983; Teichgraeber *et al.*, 1973; Willén *et al.*, 1975; Lund *et al.*, 1975) have cavity carcinoma provides useful prognostic information but has certain limitations. The single method treatment of stage I cancer carries a much poorer prognosis than was previously thought, and the incidence of microscopic cervical metastases is high (Lee *et al.*, 1972; Krause *et al.*, 1973; Teichgraeber *et al.*, 1984).

The malignancy grading system based on 4 different morphological characteristics for the tumour cell population, and 4 characteristics for the tumour–host relationship, initially used in the analysis of laryngeal cancer (Jakobsson *et al.*, 1973), has also been applied in oral cavity carcinomas. Studies on palatal, gingival, and lingual carcinomas (Eneroth *et al.*, 1973; Willén *et al.*, 1975; Lund *et al.*, 1975) have disclosed statistically significant differences in survival for patients with high and low malignancy scores. The somewhat modified malignancy grading used in lingual carcinomas by Holm *et al.* (1982) showed a correlation between the malignancy score on the one hand and *T* classification or the presence of lymph node metastases at the time of diagnosis on the other.

Both TNM classification and malignancy grading are partly subjective methods, designed to predict the behaviour of malignant lesions. DNA content, proliferative activity (S-phase), and the occurrence of polyploid nuclei are more objective estimates and yield important complements to the clinical and pathological classification of many types of tumours (Atkin, 1976; Friedlander *et al.*, 1984; Matsuura *et al.*, 1986). Studies by Holm *et al.* (1980) and Holm (1982) indicate that DNA measurements of oral cavity carcinomas may contain prognostic information.

The aims of the present investigation were to determine DNA ploidy, S-phase fraction, and the presence of polyploid nuclei, and to perform histological malignancy grading of T1 and T2 oral cavity carcinomas to detect possible correlations between these factors and clinical course.

### Materials and methods

The series comprised 50 patients (29 male and 21 female) with T1 and T2 squamous cell carcinoma of the oral cavity treated at Linköping University Hospital during the 17-year period 1967–1983. The ages ranged from 39 to 93 years (mean 67 years).

There were thirteen T1 and thirty-seven T2 tumours. Nine patients, 2 with T1 and 7 with T2 tumours had cervical lymph node metastases (N1). No patient had distant metastases.

Treatment was by surgery and/or radiotherapy (Table I). No major change in therapy was introduced during the actual period. T1 tumours are usually treated surgically but if metastatic nodes were present combined therapy with preoperative radiotherapy was administered including the nodes as for T2 tumours.

The follow-up time was from the date of diagnosis to the end of 1985.

### *DNA measurements*

Sections (50 $\mu$m) were prepared from representative tumour areas of formalin-fixed, paraffin-embedded material using the method described by Hedley *et al.*, (1983) with certain modifications (Risberg *et al.*, 1985; Franzén *et al.*, 1986*a*) making it suitable for static cytofluorometry on squamous cell carcinomas. Measurements were performed with the aid of a Leitz MPV3 cytophotometer (Ernst Leitz, Wetzlar, FRG) interfaced to a Luxor ABC microcomputer (Luxor, Motala, Sweden) and using the Fluora computer program (Bjelkenkrantz *et al.*, 1983*a*). About 300 tumour cell nuclei were measured in each specimen in a meander fashion to be sure that the same nucleus was measured only once. Lymphocytes visually identified in the specimen were used as reference cells for the DNA diploid value. In 3 preparations only 100–150 tumour nuclei were present.

### *DNA classification*

In the histograms the following characteristics were evaluated:

Correspondence: M. Tytor.
Received 2 April 1987; and in revised form, 24 June 1987.

*DNA ploidy level* The tumour stem cell peak in relation to the reference peak (lymphocytes) is defined as the DNA index.

(*a*) A stem cell peak with a DNA index of 0.85–1.15 is defined as DNA diploid if the tetraploid peak does not exceed 30% of the diploid peak (Figure 1a).

(*b*) DNA non-diploid tumours are those with a tetraploid peak > 30% of the diploid peak and those with a DNA index > 1.15 (Figures 1b, c).

*S-phase* The S-phase fraction was calculated by the computer, assuming a rectilinear distribution of the S-phase cells between the G0/1 - and G2/M peaks. In 8 DNA non-diploid tumours the S-phase value could not be estimated, because there were fewer than 100 tumour nuclei in the preparation or because there was more than one DNA non-diploid cell population. Due to the small number of nuclei measured, the tumours were classified into three groups: 1. tumours with an S-phase < 10%, 2. tumours with an S-phase 10%–20%, and 3. tumours with an S-phase > 20%.

*Polyploid nuclei* (*PPN*) Nuclei with a fluorescence value greater than 2.5 × the basal modal peak. The estimation of PPN in the tumours was qualitative.

*Histological examination and malignancy grading*

All tumours were graded histologically into well, moderately well, and poorly differentiated.

The malignancy grading was performed as described by Jakobsson *et al.* (1973). The system described and applied by Glanz and Eichhorn (1985) was also employed. According to Glanz and Eichhorn, 2 of 4 characteristics (i) differentiation and polymorphism, and (ii) structure and margins of the tumours, are related to the tumour cell population; and 2 characteristics (iii) vascular and perineural invasion, and (iv) plasmo-lymphocytic infiltration, are related to the tumour-host relationship. The gradings were performed three times by the same pathologist. There were more than 3 points difference in 4 specimens between the first and second examination. However, there was a very good concordance between the second and third examination with only 1–2 points difference in 3 specimens, and the results from the third examination were used.

*Statistical analysis*

The chi-square method was applied and the standard Kaplan–Meier method was used for calculating the cumulative survival.

**Results**

The results are listed in Tables II and III and Figures 2–5.

**Table I** Forms of treatment in relation to TNM classification

| | *T1* | | *T2* | |
|---|---|---|---|---|
| | *N0* (*11*) | *N1* (*2*) | *N0* (*30*) | *N1* (*7*) |
| External radiotherapy | 1 | – | 3 | 1 |
| Surgery | 5 | – | 2 | – |
| External radiotherapy + surgery | 2 | 1 | 17 | 4 |
| Surgery + external radiotherapy | – | – | 1 | – |
| Interstitial radiotherapy | – | – | 1 | – |
| Interstitial radiotherapy + surgery | 3 | 1 | 6 | 2 |

Twenty-eight tumours were classified as DNA diploid and 22 as DNA non-diploid. There were 12 tumours with an S-phase fraction below 10%, 20 tumours with an S-phase fraction between 10% and 20%, and 10 tumours with an S-phase fraction above 20%. The S-phase fraction could not be estimated in 8 tumours. Polyploid nuclei were found in 58% (29/50) of the tumours.

There was no difference in DNA pattern related to location of the tumours. Nine of the 24 lingual carcinomas and 13 of the remaining 26 were DNA non-diploid.

The DNA pattern and histological malignancy grading in relation to clinical stage of the tumours are shown in Table II. There was no significant correlation between T stage of the tumours and DNA ploidy or malignancy grading. However, a significant correlation existed between the presence of cervical lymph node metastases (N1) and DNA ploidy ($P < 0.01$, chi-square), and both systems of histological malignancy grading ($P < 0.05$, chi-square). The presence of polyploid nuclei did not correlate either with T-, or N-status of the tumours.

The DNA pattern and histological malignancy grading in relation to local, regional and distant recurrences, and the total number of patients with involved cervical lymph nodes (initial and late) are shown in Table III. There were no significant differences between tumour DNA ploidy, occurrence of PPN and the frequency of local, regional or distant recurrences. The occurrence of PPN, however, correlated with the presence of the total number of initial and late lymph node metastases ($P < 0.05$, chi-square). All tumours with distant metastases were DNA non-diploid and had PPN. The histological malignancy score according to both Jakobsson and Glanz and Eichhorn correlated with regional recurrences ($P < 0.01$) and with the total number of lymph node metastases (initial and late) ($P < 0.001$, chi-square). There was, however, no significant correlation between histological malignancy scores and local recurrences or distant metastases.

Twenty-four patients were given preoperative external beam radiotherapy. In 12, residual carcinoma was present in the operation specimen; 7 developed local recurrences. Sixty

**Figure 1** (a) DNA diploid tumour. The stem cell peak has a DNA index of 1. The tetraploid peak does not exceed 30% of the diploid peak. (b) DNA non-diploid tumour. The stem cell peak has a DNA index of 1. The tetraploid peak exceeds 30% of the diploid peak. (c) DNA non-diploid tumour. The stem cell peak has a DNA index > 1.15.

**Table II** Clinical stage of the tumours in relation to DNA pattern and histological malignancy grading

| | | *T1* | | *T2* | | *Tx* | |
|---|---|---|---|---|---|---|---|
| | | *N0* (*11*) | *N1* (*2*) | *N0* (*30*) | *N1* (*7*) | *N0* (*41*) | *N1* (*9*) |
| Diploid | (28) | 10 | 0 | 17 | 1 | 27 | 1 |
| Non-diploid | (22) | 1 | 2 | 13 | 6 | 14 | 8 |
| Polyploid nuclei (PPN) | (29) | 4 | 1 | 18 | 6 | 22 | 7 |
| Histological malignancy grading (Jakobsson) | | | | | | | |
| mean score | | 16.4 | 18.0 | 16.9 | 18.2 | 16.8 | 18.1 |
| ≦16 | (16) | 5 | 0 | 11 | 0 | 16 | 0 |
| >16 | (34) | 6 | 2 | 19 | 7 | 25 | 9 |
| Histological malignancy grading (Glanz & Eichhorn) | | | | | | | |
| mean score | | 5.3 | 6.0 | 5.5 | 6.1 | 5.4 | 6.1 |
| ≦5 | (16) | 5 | 0 | 11 | 0 | 16 | 0 |
| >5 | (34) | 6 | 2 | 19 | 7 | 25 | 9 |

**Table III** Relation between DNA pattern or histological malignancy grading and development of recurrences and lymph node metastases (N1 + regional recurrences)

| | | *Recurrences* | | | *Lymph node metast.* (*initial and late*) |
|---|---|---|---|---|---|
| | | *Local* | *Regional* | *Distant* | |
| Diploid | (28) | 9 | 8 | 0 | 8 |
| Non-diploid | (22) | 8 | 4 | 3 | 10 |
| Without polyploid nuclei (PPN) | (21) | 5 | 3 | 0 | 4 |
| With polyploid nuclei (PPN) | (29) | 12 | 9 | 3 | 14 |
| S-phase (%) | | | | | |
| <10 | (12) | 3 | 5 | 0 | 5 |
| 10–20 | (20) | 8 | 2 | 1 | 5 |
| >20 | (10) | 2 | 3 | 0 | 5 |
| Histological malignancy grading (Jakobsson) | | | | | |
| ≦16 | (16) | 5 | 0 | 0 | 0 |
| >16 | (34) | 12 | 12 | 3 | 18 |
| Histological malignancy grading (Glanz & Eichhorn) | | | | | |
| ≦5 | (16) | 5 | 0 | 0 | 0 |
| >5 | (34) | 12 | 12 | 3 | 18 |

seven per cent (8/12) of the patients with DNA diploid tumours and 33% (4/12) of those with DNA non-diploid tumours given preoperative radiotherapy had residual carcinoma in the operation specimen; a difference, however, not statistically significant. Patients with remaining tumour after preoperative radiotherapy showed a higher local recurrence rate (7/12; 58%) than those without (0/12; 0%) ($P<0.01$; chi-square).

Twenty-one patients (54%) died of carcinoma; 18 are alive and free from cancer after at least 39 months; 11 patients died of intercurrent disease with no sign of cancer.

The 5-year cumulative survival (Kaplan–Meier) in relation to clinical stage, DNA ploidy and malignancy grading is illustrated in Figures 2–4. Patients with nodal involvement (N1) had a statistically significant worse prognosis than those without (N0) ($P<0.01$). Patients with DNA diploid tumours had a better 5-year cumulative survival than those with DNA non-diploid tumours, but the difference was not statistically significant. Patients with high tumour histological malignancy scores had a worse prognosis than those with low scores ($P<0.001$).

There was a statistically significant difference in survival rates for patients with well differentiated tumours compared to patients with poorly differentiated tumours ($P<0.001$) and between patients with moderately well differentiated tumours and poorly differentiated ones ($P<0.05$) (Figure 5).

### Discussion

The presence of lymph node metastases is clinically the most important prognostic factor in oral cavity carcinoma (Hibbert *et al.*, 1983). This is corroborated by the present findings.

Poorly differentiated carcinomas carried a worse prognosis than well differentiated ones as was shown by Holm *et al.* (1982).

DNA measurements of squamous cell carcinomas of the head and neck region show similar DNA ploidy for tumours from various locations, and most tumours in the series reported by Holm *et al.*, (1980) and Holm (1982) were DNA non-diploid. However, most tumours in the present series were DNA diploid (56%), possibly because small tumours tend to be DNA diploid (Kaplan *et al.*, 1986; Tytor *et al.*, 1987).

In line with the findings of Holm *et al.* (1982) and Hedley *et al.* (1984) the DNA non-diploid tumours metastasized more frequently to cervical lymph node than the DNA diploid ones ($P<0.01$, chi-square).

The literature reports a worse prognosis for patients with DNA non-diploid tumours than for those with DNA diploid ones (Atkin *et al.*, 1976; Holm *et al.*, 1982; Auer *et al.*, 1984). There was a difference in our material too, albeit not statistically significant.

**Figure 2** Survival in relation to tumour stage. Life table analysis according to Kaplan–Meier.

**Figure 4** Survival in relation to malignancy grading (Jakobsson and Glanz and Eichhorn). Life table analysis according to Kaplan–Meier.

**Figure 3** Survival in relation to DNA ploidy. Life table analysis according to Kaplan–Meier.

**Figure 5** Survival in relation to histological differentiation. Life table analysis according to Kaplan–Meier.

The highest S-phase values were found in the DNA non-diploid tumours, low values occurring mainly in the DNA diploid group (cf. Johnson *et al.*, 1985). No significant differences emerged between occurrence of lymph node metastases and the S-phase values. The proliferative activity (S-phase) as a predictor of outcome has been examined in other studies (Olszewski *et al.*, 1981; Hanson *et al.*, 1982). The S-phase analyses of fresh and paraffin embedded material yielded almost identical results (Risberg *et al.*, 1985) and there was a good correlation using flow and static cytofluorometry on fresh material and static fluorometry on paraffin-embedded material (Franzén *et al.*, 1986*a*). The S-phase estimation, however, cannot be as reliable as that obtained by flow cytometry, where the number of measured nuclei is 10,000 or more. Therefore, no further conclusions are drawn from the S-phase values.

The presence of polyploid nuclei seems to be a negative prognostic factor (Greisen, 1975; Bjelkenkrantz *et al.*, 1983*b*; Olofsson *et al.*, 1986). In the present investigation, tumours without PPN were associated with a slightly better survival than those with PPN; however, not statistically significantly so.

An interesting observation is that the patients given preoperative radiotherapy and with histologically persisting tumour tissue showed a higher local recurrence rate (7/12; 58%) than those without tumour tissue (0/12; 0%) ($P<0.01$, chi-square). Concordant with the findings of Franzén *et al.* (1986*b*) the DNA non-diploid tumours given preoperative radiotherapy were more often eradicated than the DNA diploid tumours, but the difference was not statistically significant.

Malignancy grading allows multifactorial assessment of the tumour and the tumour–host relationship, and has proved valuable in predicting the clinical course in different head and neck carcinomas (Holm *et al.*, 1982). It is nevertheless a subjective method with varying reproducibility (Graem *et al.*, 1980). No correlation emerged between Jakobsson and Glanz and Eichhorn scores and the T-classification, which accords with some other reports, on lingual and gingival carcinoma (Lund *et al.*, 1974; Willén *et al.*, 1975), but contradicts the findings of Holm *et al.* (1982). The malignancy grading, however, correlated significantly with the occurrence of lymph node metastases. As claimed by Eneroth *et al.* (1973), Willén *et al.* (1975) and Holm *et al.* (1982) the total malignancy score correlated with prognosis ($P<0.001$, Kaplan–Meier).

Static cytofluorometry gives an objective evaluation of tumour ploidy and the occurrence of PPN, and correlated to lymph node metastases. DNA ploidy may also provide information about the histological response to preoperative radiotherapy (Franzén *et al.*, 1986*b*). Malignancy grading gave information both concerning lymph node metastases and prognosis.

This study was supported by the Swedish Cancer Society, Torsten and Ragnar Söderbergs Foundation and Funds from the County Council of Östergötland.

Olle Stål provided great assistance in the computer programming and Torbjörn Ledin assisted in the statistical analysis.

ATKIN, N.B. (1976). Prognostic significance of ploidy level in human tumours. Extra-uterine cancers and summary of data on 1171 tumours. *Cytobios*, **15**, 233.

AUER, G., ERIKSSON, E., AZAVEDO, E., CASPERSSON, T. & WALLGREN, A. (1984). Prognostic significance of nuclear DNA content in mammary adenocarcinomas in humans. *Cancer Res.*, **44**, 394.

BJELKENKRANTZ, K., STÅL, O. & GRÖNTOFT, O. (1983*a*). A fast reliable system for microcomputerized DNA cytofluorometry in tumour pathology. *Histochemistry*, **79**, 145.

BJELKENKRANTZ, K. LUNDGREN, J. & OLOFSSON, J. (1983*b*). Single-cell DNA measurements in hyperplastic, dysplastic and carcinomatous laryngeal epithelia, with special reference to the occurrence of hypertetraploid cell nuclei. *Anal. Quant. Cytol.*, **5**, 184.

ENEROTH, C.M. & MOBERGER, G. (1973). Histological malignancy grading of squamous cell carcinoma of the palate. *Acta Otolaryngol.*, **75**, 293.

FLETCHER, G.H. (1979). The role of irradiation in the management of squamous-cell carcinomas of the mouth and throat. *Head Neck Surg.*, **1**, 441.

FRANZÉN, G., OLOFSSON, J., RISBERG, B., KLINTENBERG, C. & NORDENSKJÖLD, B. (1986*a*). DNA-measurements on formalin-fixed, paraffin-embedded squamous cell carcinomas from different ENT-regions. *Path. Res. Pract.*, **181**, 230.

FRANZÉN, G., KLINTENBERG, C., OLOFSSON, J. & RISBERG, B. (1986*b*). DNA measurement – An objective predictor of response to irradiation? A review of 24 squamous cell carcinomas of the oral cavity. *Br. J. Cancer*, **53**, 643.

FRAZELL, E.L. & LUCAS, J.C. JR. (1962). Cancer of the tongue. Report of the management of 1554 patients. *Cancer*, **15**, 1085.

FRIEDLANDER, M.L., HEDLEY, D.W., TAYLOR, I.W., RUSSELL, P., COATES, A.S. & TATTERSALL, M.H.N. (1984). Influence of cellular DNA content on survival in advanced ovarian cancer. *Cancer Res.*, **44**, 397.

GLANZ, H. & EICHHORN, T. (1985). Die prognostische Bedeutung des histologischen 'Gradings' von Stimmlippenkarzinomen. *HNO*, **33**, 103.

GRAEM, N., HELWEG-LARSEN, K. & KEIDING, N. (1980). Precision of histological grading of malignancy sources of variation in a histological scanning system for grading cancer of the larynx. *Acta Pathol. Microbiol. Scand.*, Sect., A, **88**, 307.

GREISEN, O. (1975). Deoxyribonucleic acid content in the laryngeal mucosa with special reference to polyploid cell nuclei. *Acta Pathol. Microbiol. Scand.*, Sect. A, **83**, 704.

HANSSON, J., TRIBUKAIT, B., LEWENSOHN, R. & RINGBORG, U. (1982). Flow cytofluorometric DNA analyses of metastases of human malignant melanomas. *Anal. Quant. Cytol.*, **4**, 99.

HEDLEY, D.W., FRIEDLANDER, M.L., TAYLOR, I.W., RUGG, C.A. & MUSGROVE, E.A. (1983). Method for analysis of cellular DNA content of paraffin-embedded pathological material using flow cytometry. *J. Histochem. Cytochem.*, **31**, 1333.

HEDLEY, D.W., RUGG, C.A., NG, A.B.P. & TAYLOR, I.W. (1984). Influence of cellular DNA content on disease-free survival of stage II breast cancer patients. *Cancer Res.*, **44**, 5395.

HIBBERT, J., MARKS, N.J., WINTER, P.J. & SHAHEEN, O.H. (1983). Prognostic factors in oral squamous carcinoma and their relation to clinical staging. *Clin. Otolaryngol.*, **8**, 197.

HOLM, L.E., JAKOBSSON, P., KILLANDER, D., SILFVERSVÄRD, C. & WERSÄLL, J. (1980). DNA and its synthesis in individual tumor cells from human upper respiratory tract squamous cell carcinomas. *Laryngoscope*, **90**, 1209.

HOLM, L.E., LUNDQUIST, P.G., SILFVERSWÄRD, C. & SOBIN, A. (1982). Histological grading of malignancy in squamous cell carcinoma of the oral tongue. *Acta Otolaryngol.*, **94**, 185.

HOLM, L.E. (1982). Cellular DNA amounts of squamous cell carcinomas of the head and neck region in relation to prognosis. *Laryngoscope*, **92**, 1064.

JAKOBSSON, P.Å., ENEROTH, C.M., KILLANDER, D., MOBERGER, G. & MÅRTENSSON, B. (1973). Histologic classification and grading of malignancy in carcinoma of the larynx. *Acta Radiol. Ther. Phys., Biol.*, **12**, 1.

JOHNSON, T.S., WILLIAMSON, K.D., CRAMER, M.M. & PETERS, L.J. (1985). Flow cytometric analysis of head and neck carcinoma DNA index and S-fraction from paraffin-embedded sections: Comparison with malignancy grading. *Cytometry* **6**, 461.

KAPLAN, A.S., CALDARELLI, D.D., CHACHO, M.S. & 4 others (1986). Retrospective DNA analysis of head and neck squamous cell carcinoma. *Arch. Otolaryngol. Head Neck Surg.*, **112**, 1159.

KRAUSE, C.J., LEE, J.G. & MCCABE, B.F. (1973). Carcinoma of the oral cavity. A comparison of therapeutic modalities. *Arch. Otolaryngol.*, **97**, 354.

LEE, J.G. & LITTON, W.B. (1972). Occult regional metastasis: Carcinoma of the oral tongue. *Laryngoscope*, **82**, 1273.

LUND, C., SÖGAARD, H., ELBRÖND, O., JÖRGENSEN, K. & ANDERSEN, A.P. (1975). Epidermoid carcinoma of the tongue. Histologic grading in the clinical evaluation. *Acta Radiol. Ther. Phys. Biol.*, **14**, 513.

MATSUURA, H., SUGIMACHI, K., UEO, H., KUWANO, H., KOGA, Y. & OKAMURA, T. (1986). Malignant potentiality of squamous cell carcinoma of the esophagus predictable by DNA analysis. *Cancer*, **57**, 1810.

OLOFSSON, J., FRANZÉN, G. & LUNDGREN, J. (1986). Hypertetraploid cells in vocal cord epithelia. *Clin. Otolaryngol.*, **11**, 345.

OLSZEWSKI, W., DARZYNKIEWICZ, Z., ROSEN, P.P., SCHWARTZ, M.K. & MELAMED, M.R. (1981). Flow cytometry of breast carcinoma: Relation of tumor cell cycle distribution to histology and estrogen receptor. *Cancer*, **48**, 985.

RISBERG, B., STÅL, O., BJELKENKRANTZ, K. & 4 others (1985). Use of formalin fixed, paraffin embedded tumours for estimation of cellular DNA content and S-phase fraction by static cytofluorometry. *Acta Radiol. Oncol.*, **24**, 537.

TEICHGRAEBER, J.F. & CLAIRMONT, A.A. (1984). The incidence of occult metastases for cancer of the oral tongue and floor of the mouth: Treatment rationale. *Head Neck Surg.*, **7**, 15.

TYTOR, M., FRANZÉN, G. & OLOFSSON, J. (1987). DNA pattern in oral cavity carcinomas in relation to clinical stage and histological grading. *Path. Res. Pract.*, **182**, 202.

UICC (UNION INTERNATIONALE CONTRE LE CANCER). (1978). *TNM classification of malignant tumours* (Oral cavity. Classified 1973. Confirmed 1978), Geneva.

WILLÉN, R., NATHANSON, A., MOBERGER, G. & ANNEROTH, G. (1975). Squamous cell carcinoma of the gingiva. Histological classification and grading of malignancy. *Acta Otolaryngol.*, **79**, 146.